# Use of the self-organising map network (SOMNet) as a decision support system for regional mental health planning

**DOI:** 10.1186/s12961-018-0308-y

**Published:** 2018-04-25

**Authors:** Younjin Chung, Luis Salvador-Carulla, José A. Salinas-Pérez, Jose J. Uriarte-Uriarte, Alvaro Iruin-Sanz, Carlos R. García-Alonso

**Affiliations:** 1 Faculty of Engineering & Information Technologies, The University of Sydney, 1 Cleveland Street, Darlington, NSW 2008 Australia; 2 Department of Quantitative Methods, Universidad Loyola Andalucía, C/Escritor Castilla Aguayo, 4, 14004 Córdoba, Spain; 3 Faculty of Health Sciences, The University of Sydney, 94 Mallett Street, Camperdown, NSW 2050 Australia; 4 ANU College of Health and Medicine, Australian National University, 63 Eggleston Road, Acton, ACT 2601 Australia; 5 PSICOST Research Association, Universidad Loyola Andalucía, C/Energía Solar, 1 Edificio G, 41014 Sevilla, Spain; 6 Bizkaia Mental Health Services, Osakidetza-Basque Health Service, Biocruces Health Research Institute, Calle Maria Diaz de Haro, 58, 48010 Bilbao, Spain; 7 Gipuzkoa Mental Health Services, Osakidetza - Basque Health Service, Biocruces Health Research Institute, Paseo Doctor Beguiristain, 115, 20014 San Sebastian, Spain

**Keywords:** Mental health system, Evidence-informed policy planning, Decision support systems, Health systems engineering, Expert knowledge, Interactive visual data mining, Self-organising map network, Expert-based collaborative analysis, Key performance indicator

## Abstract

**Background:**

Decision-making in mental health systems should be supported by the evidence-informed knowledge transfer of data. Since mental health systems are inherently complex, involving interactions between its structures, processes and outcomes, decision support systems (DSS) need to be developed using advanced computational methods and visual tools to allow full system analysis, whilst incorporating domain experts in the analysis process. In this study, we use a DSS model developed for interactive data mining and domain expert collaboration in the analysis of complex mental health systems to improve system knowledge and evidence-informed policy planning.

**Methods:**

We combine an interactive visual data mining approach, the self-organising map network (SOMNet), with an operational expert knowledge approach, expert-based collaborative analysis (EbCA), to develop a DSS model. The SOMNet was applied to the analysis of healthcare patterns and indicators of three different regional mental health systems in Spain, comprising 106 small catchment areas and providing healthcare for over 9 million inhabitants. Based on the EbCA, the domain experts in the development team guided and evaluated the analytical processes and results. Another group of 13 domain experts in mental health systems planning and research evaluated the model based on the analytical information of the SOMNet approach for processing information and discovering knowledge in a real-world context. Through the evaluation, the domain experts assessed the feasibility and technology readiness level (TRL) of the DSS model.

**Results:**

The SOMNet, combined with the EbCA, effectively processed evidence-based information when analysing system outliers, explaining global and local patterns, and refining key performance indicators with their analytical interpretations. The evaluation results showed that the DSS model was feasible by the domain experts and reached level 7 of the TRL (system prototype demonstration in operational environment).

**Conclusions:**

This study supports the benefits of combining health systems engineering (SOMNet) and expert knowledge (EbCA) to analyse the complexity of health systems research. The use of the SOMNet approach contributes to the demonstration of DSS for mental health planning in practice.

**Electronic supplementary material:**

The online version of this article (10.1186/s12961-018-0308-y) contains supplementary material, which is available to authorized users.

## Background

Planners and policy-makers face complex decisions that require a deep knowledge of health systems, which involve inherently complex interactions between its structures, processes and outcomes, among multiple agents. These systems are characterised by nonlinearity, interconnectivity, self-organisation, constant change, variability and uncertainty [1]. Such complexity of health systems is even greater in mental health [2, 3]. Data for health systems can be described according to their context (e.g. socio-demography), care delivery (e.g. services, places, beds and human resources), resource utilisation (e.g. throughput activity and interventions), costs and consequences (e.g. mortality, health status, funding and quality of life adjusted years) [4]. There are increasing alternatives to collect, synthesise and represent these data in a meaningful way to allow national and international comparisons from different perspectives and stakeholders (consumer, provider, societal and government). Mental health agencies collect a vast amount of these data. However, such data collection efforts have not been accompanied by a global collaborative commitment to developing decision support systems (DSS) for resource planning and allocation. Consequently, healthcare policy faces a major health information waste that adds to the existing health systems research waste [5–7]. The effective reduction of this research waste should consider the complexity of mental health systems.

**Fig. 1:**
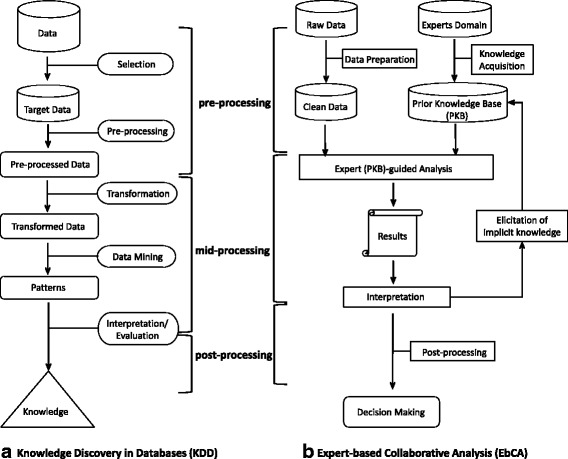
Three different phases of data processing (pre-, mid- and post-processing) for decision support systems are compared between knowledge discovery in databases (KDD) and ‘Expert-based Collaborative Analysis’ (EbCA). **a** The process of KDD [13]. **b** The operational process in EbCA [11]

Under these circumstances, DSS should incorporate all relevant sources of scientific knowledge, including observational, contextual and expert knowledge, as well as systematic reviews of experimental studies [8]. To fit this broader scope, classical ‘evidence-based health care’ has evolved into ‘evidence-informed policy’ through the addition of routine big data and local context information [9, 10], and into ‘knowledge-guided policy’ through the incorporation of domain experts to the data analysis process and to the development of DSS [11]. The relevance and practicality of incorporating expert knowledge into data processing has been approached from different perspectives, including the new framework of scientific knowledge for health systems research [12], the role of knowledge discovery in databases (KDD) [13] and DSS in mental health systems [3], and the operational model for integrating domain experts into the data analysis process (expert-based collaborative analysis (EbCA)) [11, 14]. The EbCA approach expands and systematises expert roles and tasks in the different phases of data analysis (pre-processing, mid-processing and post-processing) based on KDD (Fig. 1). This approach has been used to conduct context analysis and to produce local Atlases of Mental Health Care. These Atlases are basic tools for developing DSS in practice, providing bottom-up information on service availability, placement and workforce capacity, and resource utilisation in small catchment areas that are aggregated to the regional or national level [15–17]. The information on the context of the provision is then combined with resource utilisation data to perform systems analytics.

In previous systems analytics studies, small mental health catchment areas in Spain were analysed using hierarchical clustering [18], hierarchical Bayesian models [19], and Monte Carlo Data Envelopment Analysis (Monte Carlo Data Envelopment Analysis (DEA)) [11, 20]. The impact of these approaches in policy and planning has been extensively revised with regional and national planners in Spain. Even though these approaches have contributed to increasing the organisational knowledge by reducing uncertainty about the existing mental health systems, they showed some limitations in their use for effective DSS. The hierarchical clustering and Bayesian approaches to health systems analysis do not readily incorporate expert formal knowledge in the mid-processing of data as a rule. Therefore, the role of domain experts is limited to contributing to the establishment of the prior knowledge base at the pre-processing phase and to the implementation knowledge in the post-processing phase of KDD (Fig. 1). Based on the EbCA approach, domain experts were incorporated in the mid-processing phase using the Monte Carlo DEA model in our previous study [20]. A full system analysis was performed by generating different combinations of inputs and outputs in 27 non-correlated scenarios for Catalonia. However, this approach required the incorporation of a significant number of assumptions into the model, and the interpretation of the results by the domain experts was difficult unless extensive guidance was provided by the health systems engineer. Additionally, the Monte Carlo DEA, as well as the other above approaches, could only analyse a limited number of indicators and areas. These methods were also unable to incorporate appropriate visual tools as a component of DSS for the easy understanding of information by domain experts.

An alternative is to combine artificial neuronal networks (ANNs) with advanced information visualisation techniques in a single DSS. Based on the demand, the aim of this study is to assess a new DSS model using a novel approach, a self-organising map network (SOMNet) [21, 22], combined with the EbCA [11] for the use of knowledge-guided mental health planning. The SOMNet was developed to facilitate interactive visual data mining of complex data to enable domain experts to (1) generate and verify hypotheses; (2) express interest through the process of KDD; (3) enhance information transferring between analysts and decision-makers; (4) specify information processing and present outcomes of analytical reasoning processes; and (5) identify hidden information and elicit tacit knowledge that can be formalised and transformed into rules for further data analysis [23–25].

This paper investigates the usability of the SOMNet approach combined with the EbCA approach in developing a DSS model for analysing complex mental health systems in a real-world context. This combined DSS model is applied to the assessment of the mental health care systems of three regions in Spain. The study aims to reduce uncertainty by increasing the expert knowledge of the systems through (1) the identification of outlier areas based on the systems analysis of key performance indicator (KPI) information; (2) the identification of the distinctive global and local patterns of healthcare across the different systems; and (3) the identification of the relationships between the KPIs in the systems. The domain experts are involved in the analytical process by interpreting and evaluating the analytical results. The study then evaluates the feasibility and technology readiness level (TRL) [26, 27] of this DSS model (refer to Additional file 1 for information on the model validation process).

## Methods

This study was conducted by an international core team working in collaboration with a group of Spanish domain experts. The core development team included two systems engineers (the SOMNet developer and a knowledge engineer) and two domain experts (a health geographer and a health research clinician). The second evaluation group was composed of 13 domain experts who are end-users of the DSS, including health service researchers (*n* = 6) and health planners from the agencies of Catalonia (*n* = 3), Biscay (*n* = 2) and Gipuzkoa (*n* = 2).

### Study setting

The mental health system in Spain has been described elsewhere [28]. In Spain, there is nearly universal access to healthcare, fully devolved to 17 regions or ‘autonomous communities’ since the middle of the 1980s. Mental health care is organised in small catchment areas coordinated by a reference community centre of mental health. Typically, there is a reference acute hospital for every three to four community centres. Administrative utilisation data from the acute hospitals and community centres are representative of the use of the care services by the population in every small catchment area.

The Basque Country and Catalonia are considered benchmark regions of integrated healthcare for chronic illnesses in Europe [29, 30]. Health planning in Catalonia (7,555,830 inhabitants) depends on a single regional health department separated from the public agency for health provision. The Basque Country has a joint agency for health provision and planning, but the regional policy is devolved to the three provinces of this autonomous community – Biscay (1,148,302 inhabitants), Gipuzkoa (719,282 inhabitants) and Araba (326,574 inhabitants). Since the regional Atlas of Mental Health Care in Araba was not available at the time of analysis, this study was set with the three health systems of Catalonia, Biscay and Gipuzkoa for macro-level analysis. A total of 106 small mental health catchment areas in Catalonia (74 areas), Biscay (19 areas) and Gipuzkoa (13 areas) was set for meso-level analysis in this study (see Additional files 2 and 3 for the area label and system map information).

### Data sources

A minimum metadata set (MMS) was developed for the data analysis based on the main sources of information described below.

#### The Integrated Atlases of Mental Health Care

These Atlases have been developed by the PSICOST Research Association using the DESDE-LTC standard classification system (Additional file 4). The DESDE-LTC identifies and codes functional care teams, called ‘Basic Stable Inputs of Care’, based on their principal activity or main types of care, namely hospital/residential care, day care, outpatient care, accessibility to care, self-help and voluntary care, and information for care. The DESDE-LTC system and its usability for international comparison of service provision have been extensively described [31–33]. The Atlases provide comprehensive information on the three regional health systems (Catalonia [16, 34], Biscay [35] and Gipuzkoa [36]). This information describes the boundaries and subsystems (defined by small catchment areas), the social and demographic characteristics relevant for mental health planning, and the provision of healthcare for the target population with a mental disorder by regions and catchment areas. It includes (1) the availability of all the services provided by every sector (health, social, employment, education and justice); (2) the placement capacity which comprises all beds (hospital and residential care) and places in day care by catchment area; and (3) the workforce capacity measured by full time equivalents of the professional staff by service, catchment area and region [28].

#### Regional health databases

Census data from the National Institute of Statistics were used to compile the social and demographic characteristics. The regional health databases were accessed to obtain administrative prevalence and incidence of mental disorders in the three systems and the resource utilisation data, which includes the number of visits in community mental health centres, hospitalisation rates, length of stay and readmission rates. The utilisation data of acute hospital and outpatient care were used in Catalonia (provided by the Catalan Department of Health) and the Basque Country (provided by the Biscay and Gipuzkoa Mental Health Services). The utilisation data of day care was not included due to missing data, lower reliability and absence of data linkage in the database at the time of the data analysis.

### Procedure

The EbCA approach [11] was used for the incorporation of domain experts into several stages of the KDD process (pre-, mid- and post-processing of data). The EbCA also provides an extension of the operational description of the mid-processing phase of KDD (data transformation, data mining and result interpretation). It is structured by refining the explicit prior expert knowledge base that is used in data analysis by transforming implicit knowledge into formal rules that are incorporated in the mid-processing of data following an iterative process (Fig. 1).

#### Pre-processing phase: database development based on the EbCA

The pre-processing phase comprised five steps involving a core team of knowledge engineers and systems researchers working with different groups of domain experts in health service research and policy planning at local, regional and national levels in Spain. The details of each step have been previously published [4, 16, 20, 37].

In summary, the first step developed the basic model of community mental healthcare. This model was developed by a group of health service researchers using the data from 12 representative small catchment areas in Spain [37]. The second step was the development of a taxonomy of indicators for health systems research in Spain that could be applied at local, regional and national levels. This typology was developed with a group of health service researchers, national health planners and other stakeholders [4]. The third step was the selection of KPIs for regional health systems analytics based on the former typology and the information provided by the Atlases of Mental Health Care [16]. A group of health service researchers, planners and managers from Catalonia selected a reduced minimum set of 64 KPIs for service availability, placement capacity, workforce capacity, resource utilisation and costs. The fourth step composed metadata by clustering KPIs in meaningful sets and defining adequate value ranges of the KPIs (e.g. the adequate range of ‘length of stay’ is between 16 and 21 days for acute hospital care in urban areas). The KPIs were then grouped into different categories (datasets) for inputs and outputs. The datasets were used for producing input and output scenarios to analyse the relative technical efficiency of small catchment areas in Catalonia [20]. In the last step, knowledge on the data of Catalonia was transferred to the Basque Country. The metadata of Catalonia was presented to a group of mental health planners in the two provinces (Biscay and Gipuzkoa) of the Basque Country with visual information of the care provided in these two regions obtained from the Atlases of Mental Health Care. This information was used by the planners to provide their regional list of the KPIs and ranges.

From the pre-processing of data, the domain experts of the three regions agreed on a common list of KPIs. This study’s core development team incorporated this information into a MMS for the data analysis. As seen in Table 1, the MMS was arranged in three input datasets, namely service availability (AVA) using 12 indicators, placement capacity (PLA) using 13 indicators and workforce capacity (WOF) using 33 indicators, and one output dataset, namely resource utilisation (USE), using 6 indicators. Since the MMS is composed of the KPIs from different categories, it is significant to analyse the system patterns and behaviours within each dataset and between different datasets (e.g. inputs and outputs) to obtain integrated bottom-up information of the healthcare systems and subsystems (small catchment areas).

**Table 1:** The minimum metadata set (MMS) for the data analysis in this study. The selected 64 key performance indicators (KPIs) of mental healthcare are divided into three input datasets (Service Availability (AVA, labelled as ‘A’), Placement Capacity (PLA, labelled as ‘P’) and Workforce Capacity (WOF, labelled as ‘W’)) and one output dataset (Resource Utilisation (USE, labelled as ‘U’)). The KPIs in each dataset are organised based on the main type of care and the DESDE-LTC classification system [34]

Indicators (64)	Input (12)	Input (13)	Input (33)	Output (6)
Main type of care	AVA (No. per 100,000 IH) clinical teams	PLA (No. per 100,000 IH) beds and places	WOF (No. per 100,000 IH) full-time equivalents	USE
Hospital and residential care				
Acute hospital care, e.g. acute ward	A1	P1	W1 (psychiatrists)	U1 (discharge)^a^
			W2 (psychologists, nurses)	U2 (length of stay)^b^
			W3 (total professionals)^d^	U3 (re-admission)^c^
Non-acute hospital care, e.g. sub-acute ward	A2	P2	W4 (psychiatrists)	–
			W5 (psychologists, nurses)	
			W6 (total professionals)	
Non-acute non-hospital care, e.g. non-acute crisis home	A3	P3	W7 (psychiatrists)	–
			W8 (psychologists, nurses)	
			W9 (total professionals)	
High intensity residential care, e.g. hostel	A9	P8	W24 (psychiatrists)	–
			W25 (nurses)	
Residential care (others), e.g. supported accommodation/group homes	A10	P9	W14 (psychiatrists)	–
24-h medical support hospital and residential care	–	P13	W31 (total professionals)	–
24-h medical support non-acute hospital and residential care	–	–	W32 (psychiatrists)	–
			W33 (total professionals)	
Residential care	A4	P4	W10 (psychiatrists)	–
			W11 (psychologists)	
			W12 (nurses)	
			W13 (total professionals)	
Day care				
Acute health day care, e.g. day hospital	A5	P5	W14 (psychiatrists)	–
			W15 (psychologists, nurses)	
			W16 (total professionals)	
Work-related day care, e.g. social firm/enterprise	A11	P11	W29 (total professionals)	–
Non-acute health day care, e.g. day health centre	A6	P6	W17 (psychologists)	–
			W18 (total professionals)	
Day care (others), e.g. social club	A7	P7	W19 (total professionals)	–
Acute and non-acute health day care	–	P10	W26 (psychologists)	–
			W27 (nurses)	
			W28 (total professionals)	
Non-health day care	A12	P12	W30 (total professionals)	–
Outpatient care				
Non-acute non-mobile outpatient care, e.g. outpatient care centre	A8	–	W20 (psychiatrists)	U4 (treated prevalence)^a^
			W21 (psychologists)	U5 (treated incidence)^a^
			W22 (nurses)	U6 (frequency)^a^
			W23 (total professionals)	

^a^The measured value unit is No. per 1000 IH

^b^The measured value unit is No. of days

^c^The measured value unit is No. per 100 discharges

^d^W3 = W1+W2+other professionals (e.g. occupational therapists, care assistants and social workers)

*No.* number, *IH* inhabitants

#### Mid-processing phase: interactive visual data mining and interpretation using the SOMNet

The MMS information on the four datasets of 106 small catchment areas in the three health systems (Catalonia, Biscay and Gipuzkoa) was used to assess integrated mental healthcare provision and resource utilisation. The core team developed a DSS model that uses the SOMNet and the incorporation of the experts in the mid-processing of the data based on the EbCA.

**Fig. 2:**
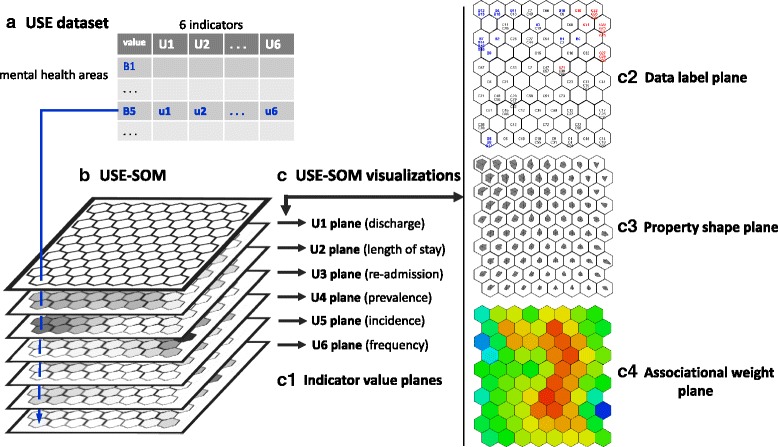
A self-organising map (SOM) example using the resource utilisation (USE) dataset. **a** The USE dataset table with six indicators (column) for 106 mental health areas (row). **b** The USE-SOM created by learning the USE dataset. **c** The visualisation examples of the USE-SOM. (c1) The indicator value planes underneath the USE-SOM. A mental health area, B5 is located on a neuron of the USE-SOM where its six indicator value locations are the same through the planes. (c2) The mental health areas are interpolated into the USE-SOM. The area labels are coloured in blue for Biscay, red for Gipuzkoa and black for the Catalonia areas. (c3) The property shape plane of the USE-SOM. A shape in a neuron represents the six indicator values of the neuron. The star glyph shape is created by marking the indicator values on the six evenly angled branches from the centre. (c4) A weight distribution is visualised on the USE-SOM. If a neuron has high weight its colour is red, while a low weight is indicated in blue

A self-organising map (SOM) is an ANN, which is a parallel information process paradigm inspired by the biological nervous system [38, 39]. ANNs learn complex nonlinear relations directly from the data being modelled instead of using a set of instructions of conventional computation. ANNs have a broad range of successful applications, including pattern recognition and intelligent search [40]. As an ANN, the SOM learns a high-dimensional data space (e.g. Fig. 2a) based on the data similarity and projects it onto a low-dimensional grid map space (e.g. two-dimensional hexagonal grid map in Fig. 2b). A set of ‘neurons’ in the map are topologically organised and adaptively represent the original data space by reflecting the data properties. Thus, the SOM has been useful to learn complex patterns based on the topological information within only a single dataset. The details of the SOM algorithm and its visualisation can be found in the work of Kohonen [39], and the visualisation examples of the USE dataset are shown in Fig. 2c1–4.

Based on machine learning and information visualisation capabilities of the SOM, the SOMNet is developed for interactive visual data mining between multiple datasets [21, 22]. The SOMNet learns the structural relationships between different datasets by its weight association. It also incorporates various visualisation methods (Fig. 2) that enable domain expert interactive pattern recognition by processing and interpreting analytical information. The functional structure of the SOMNet is described as follows:

- Data formation and networking: The SOMNet facilitates the selection and normalisation of input and output datasets to be networked from a given database. These datasets are used for handling missing data, analysing outliers and exploring data patterns within a dataset and between datasets.
- Interactive visual data mining: The SOMNet incorporates an automated machine learning process, information visualisation and user interaction tools in analysing data and obtaining relevant information. The analytical process is iterative until knowledge-based information is obtained for decision support.
- Analytical interpretation: The SOMNet enables expert knowledge incorporation into the data mining for evaluating analytical processes and results. Based on the visual information processing, it also enables domain experts exploring and guiding other steps of data analysis for eliciting tacit knowledge.

**Fig. 3:**
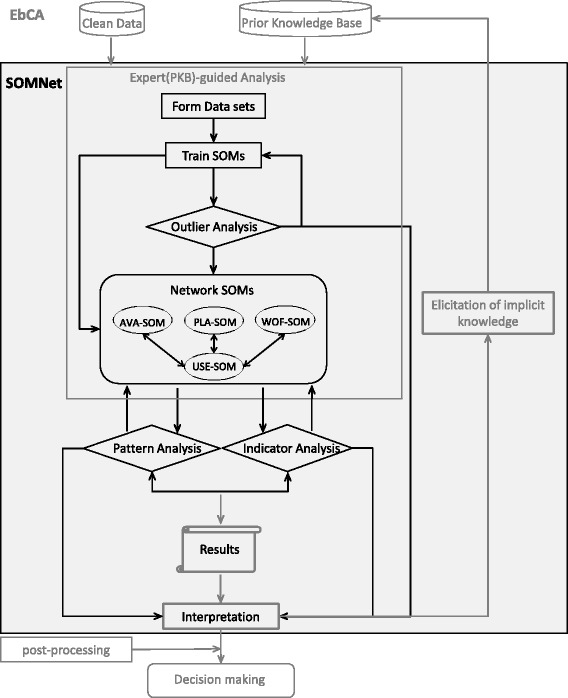
The SOMNet analysis procedure applied to this study based on the EbCA process. The partial EbCA is shown (the full version is in Fig. 1b). The SOMNet process is indicated in black in the grey shaded area of the mid-processing phase of the EbCA. The analytical processes of the SOMNet are iterative until the analytical goals are achieved for knowledge discovery

Based on the functional design of the SOMNet, expert knowledge can be incorporated in the mid-processing phase of KDD. The SOMNet approach allows expert knowledge to be combined with the EbCA from the prior knowledge base or expert-guided data analysis for result interpretation (Fig. 3).

There are three main goals defined for the SOMNet application, namely (1) system outlier identification (small catchment area outliers), (2) system pattern identification (global and local patterns) and (3) KPI relationship identification (input and output indicator patterns). The domain experts in the core team iteratively evaluate the analytical functions and results provided by the SOMNet through the reasoning process.

#### Post-processing phase: the feasibility and TRL assessment of the DSS model

The information on the analytical processes and results of the SOMNet was presented to a group of 13 domain experts. They had previous expertise on health systems research and planning, on the characteristics of the reference study regions, and on the use of other tools and methods for decision-making in mental health. Therefore, they were able to evaluate the usability of the SOMNet approach compared with other approaches used in the previous analysis for DSS. The DSS model using the SOMNet approach was validated by the domain experts using a feasibility checklist and the previous experience in the use of operations research models, and visualisation tools (the Monte Carlo DEA for relative technical efficiency analysis [20] was used as the main comparator). The feasibility checklist had 12 items grouped in four dimensions, namely ‘applicability’, ‘acceptability’, ‘practicality’ and ‘feasibility efficiency’ [41]. Additionally, two more questions referred to the ‘novelty’ and ‘potentiality’ of the SOMNet approach for its implementation. A value range [0, 10] from ‘low’ to ‘high’ was used in the checklist. The checklist information with the formal definition of the items is available in Additional file 1.

The domain experts provided their feedback based on their knowledge increase and refinement using the different approaches for DSS at this post-processing phase. In addition to this comparative feasibility evaluation, the TRL [26, 27] of the DSS model for mental health planning in practice was assessed by the core team in collaboration with the regional coordinators of this study. The results obtained from the feasibility evaluation were used for this appraisal.

## Results

We describe the study results in two parts, firstly the analytical results of the SOMNet application and, secondly, the results of the DSS feasibility evaluation using the SOMNet.

### Analytical results

We described the SOMNet functionality based on the expert interpretation of the analytical results in achieving the main goals given in this study. The analytical results obtained for each of the three goals are described below.

#### System outlier identification

In the SOMNet, a preliminary projection of the data space with 106 areas from the three mental health systems was conducted for the system outlier analysis. The four initial SOMs (AVA-SOM, PLA-SOM, WOF-SOM and USE-SOM) were trained for the four datasets categorised from the MMS by normalising the KPI values.

**Fig. 4:**
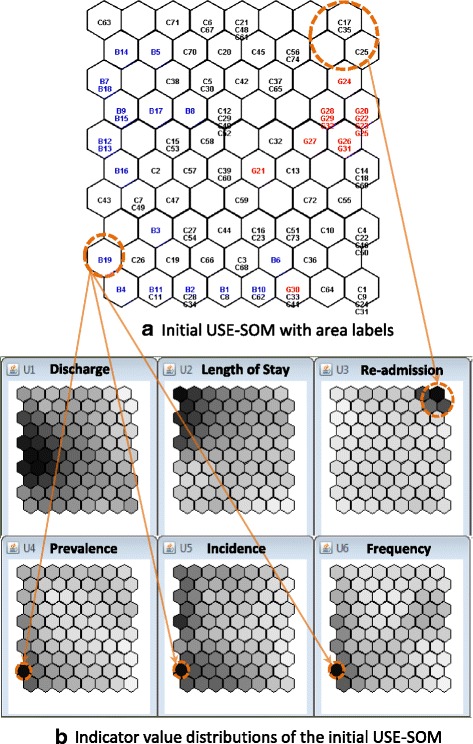
The visual identification of the system outliers in the initial USE-SOM. **a** The initial USE-SOM with the small mental health areas labelled (black, blue and red for Catalonia, Biscay and Gipuzkoa areas, respectively). The identified outlier areas are circled in orange. **b** The six indicator value planes of the USE-SOM showing the extreme values of the circled areas. The darker grey colour indicates the higher indicator value

In the AVA-SOM, three small mental health areas (C63, C64 and C74) in Catalonia were identified as outliers with extremely low or high values for the availability indicators. They were interpreted as remote and isolated areas having different service characteristics from the other areas in Catalonia. They are also distant from the reference service facilities (more than 100 km) with a very low population. These areas could be reasonably removed. Another three mental health areas (C17, C25 and C35) in Catalonia were identified as outliers in the USE-SOM with an extremely high value for the utilisation indicator, U3 (readmission) as seen in Fig. 4b. The U3 pattern over the map space is flattened by the effect produced by these three areas. A Biscay area, B19, was also identified as an outlier due to its extremely high value for the utilisation indicators of U4 (treated prevalence), U5 (treated incidence) and U6 (frequency) (Fig. 4b). B19 was interpreted as an area mainly managed in private clinics working under contract with the public system and was also removed from the systems analysis after the revision made by the domain experts. The outlier areas of C17, C25 and C37 in Catalonia are managed by the regional public health administration and are in urban areas highly connected with the rest of the system. Due to their relevance, they were kept in this study, but their extreme values for U3 were adjusted to the 97.5th percentile of the highest value from the other areas.

Through the pattern examination and interpretation, the system outliers were identified and processed to avoid pattern distortion by accounting for their relative impacts on the systems, particularly in relation to their connectivity with the other local systems. After processing the outliers, the four datasets were newly formed with a total of 102 small catchment areas. The mental health system patterns and indicators were then analysed using the newly trained and networked SOMs in the SOMNet.

#### System pattern identification and explanation

**Fig. 5:**
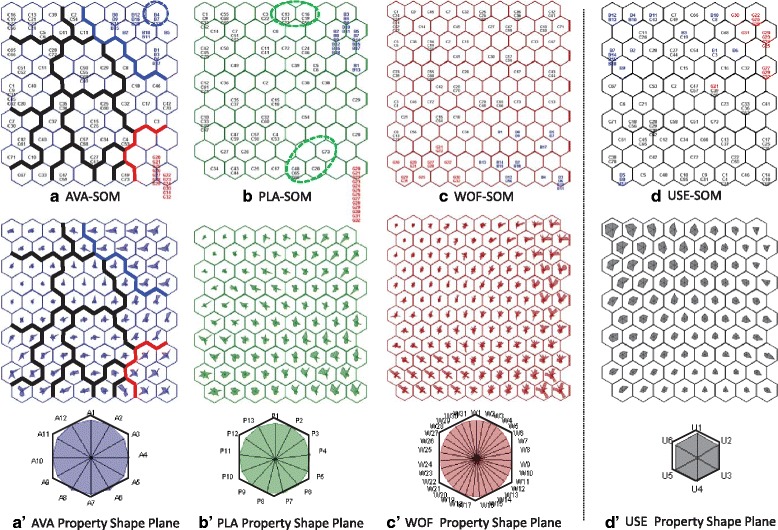
The visual identification of the global and local patterns of the mental health systems in Spain. The input SOMs are in **a** AVA-SOM, **b** PLA-SOM and **c** WOF-SOM, and the output SOM is in **d** USE-SOM. Their data property shape planes are visualised in (a'), (b'), (c') and (d'), respectively. The corresponding indicator values are pointed on the evenly angled star branches and connected to yield the shape. The legend of the property shape for each SOM is given with the order of indicators shown clockwise

The mental health system patterns were first identified at the global level (macro-level analysis of three systems). The input SOMs for availability (AVA-SOM), placement (PLA-SOM) and workforce capacity (WOF-SOM) are represented in Fig. 5a, b and c, respectively. These patterns show clear boundaries for the property dissimilarity among the systems. As seen in Fig. 5a', b' and c', the mental health areas in a set of neighbouring neurons were found as a cluster showing similar properties. The global clusters in the AVA-SOM are exemplified in Fig. 5a (drawn by the system colour lines), where the areas in Biscay are very similar to each other but very different from those in Gipuzkoa, highlighting the public service management system in the Basque Country. However, the areas in Catalonia are topologically dispersed over the maps without mixing with the areas in Biscay and Gipuzkoa, showing both public and private management types under an agreement with the regional public health administration.

The small mental health area patterns were also visually analysed at the local level (meso-level analysis of 102 catchment areas). The proximity of the SOM neurons indicates the areas having similar properties. As the local information was observed by the property similarity over the SOM, a neuron with more than one area was used as a unit cluster showing the closest property. For the input SOMs, the areas in Biscay show very similar properties as they are topologically very close to each other. The areas in Gipuzkoa also show very similar properties, while the areas in Catalonia have a more diverse pattern distribution over the region. Based on the topological property information, the further local clusters of Catalonia are exemplified in the AVA-SOM (Fig. 5a), which showed the degree of property dissimilarities across different local clusters and areas. However, the output USE-SOM (Fig. 5d and d') shows that the areas of the systems are mixed over the map space, indicating the utilisation variations for the similar resource property due to the different factors and characteristics of context [10].

The domain experts visually examined and explained the integrated global and local pattern information using the indicator value or property shape planes of the SOMs. From the availability AVA-SOM (Fig. 5a and a'), the differences in the pattern of availability were clearly captured by the higher values for A10 (residence care others) in Biscay and the higher values for A9 (high intensity residential care) in Gipuzkoa, where it has more long-stay hospitals. The local areas in Biscay were also compared with the value variation of A8 (non-acute non-mobile outpatient care). The rates of A8 for the circled areas (B4, B7 and B13) were higher than the other areas in the system since they have a very low population density. In relation to the placement PLA-SOM (Fig. 5b and b'), the areas in Biscay show the four unit clusters while the areas in Gipuzkoa show only one unit cluster. This was explained by geographical zoning in four acute hospital areas in Biscay (Basurto, Cruces, Galdakano and Zamudio hospitals) and one in Gipuzkoa (Donostia hospital). The local cluster with the areas of C13, C21, C23, C16, C19 and C56 circled in Catalonia show that their placement capacity properties are similar to the urban areas in Biscay. However, the local cluster with the areas of C20, C48, C65, C66 and C73 in Catalonia show similar patterns to the areas in Gipuzkoa. These small areas are covered by a reformed large psychiatric hospital (Pere Mata hospital) with a similar pattern of care in the predominantly rural areas in Gipuzkoa. The areas in Biscay show different patterns while the areas in Gipuzkoa have very similar patterns of the workforce WOF-SOM (Fig. 5c and c'). This was explained by the same pattern information of the placement in the PLA-SOM. From the utilisation USE-SOM (Fig. 5d and d'), the patterns do not show clear boundaries between the different systems, and the clusters are mixed with the different system areas. The areas in Biscay show many isolated patterns for resource utilisation. In Gipuzkoa, the area, G21 shows a different utilisation property with the higher values of U1 (discharge) and U3 (re-admission) than the other areas in the system, which was explained by the high rate of older people living alone in this area.

Using the SOMNet, mental health systems were analysed by providing meaningful pattern information based on the system property dissimilarity in each dataset. Different patterns of mental health care provisions in the three different systems (Catalonia, Biscay and Gipuzkoa) were identified at a global level. The local patterns of the small mental health areas in the systems were also simultaneously examined by capturing the relative pattern difference between them. The small mental health areas showed distinctive pattern information between the input datasets and the output dataset. The domain experts who participated in the SOMNet analysis process extracted the integrated bottom-up pattern information from the complex system data. Such pattern information, which was difficult to capture in previous studies, was visually identified, interpreted and evidentially justified in the DSS model using the SOMNet.

#### KPI relationship identification and interpretation

In the SOMNet, the input SOMs of availability, places and beds, and workforce (AVA-SOM, PLA-SOM and WOF-SOM) were networked to the output SOM of utilisation (USE-SOM), since the whole data space represents the association between the multiple datasets. Once the structure of each dataset was analysed, the structures between input and output datasets were analysed using their weight association. Using the SOMNet, the five input resource KPIs of acute hospital care were analysed for their ideal relationships to the output utilisation KPIs. The desired values of the five input KPIs (A1 (service availability), P1 (placement capacity), W1 (the number of psychiatrists), W2 (the number of psychologists and nurses) and W3 (the number of total professionals)) and the ideal values of the three output KPIs (U1 (discharge), U2 (length of stay) and U3 (readmission)) were provided by the prior expert knowledge, obtained from the previous EbCA studies (Table 2).

**Table 2:** The input- and output-driven analyses of indicators for acute hospital care. The indicator values are provided (A) by the expert knowledge and estimated (B) by the SOMNet. The SOMNet estimations for the output-driven and new input-driven analysis of the indicator, W1 (the number of psychiatrists), are also provided. The values of the indicators are qualitatively categorised from low to high for their quantitative value range from minimum (min) to maximum (max) in the form of [min–max] using a 20% interval

Input indicator			Analysis	Output indicator		
				discharge (*U1*) [0.41–5.88] (A) 2.50 low-medium	length of stay (*U2*) [11.89–34.23] (A) 19.00 low-medium	re-admission (*U3*) [0.00–36.88] (A) 10.00 low-medium
Acute hospital care	*A1*	[0.16–0.89]				
Availability		(A) 0.35	input-driven	(B) 2.59	(B) 17.74	(B) 10.75
		low-medium	→	low-medium	low-medium	low-medium
Acute hospital care	*P1*	[7.13–26.68]				
Placement		(A) 12.50	input-driven	(B) 2.46	(B) 18.75	(B) 10.79
		low-medium	→	low-medium	low-medium	low-medium
Acute hospital care	*W1*	[0.16–5.23]				
Psychiatrists		*(A) 3.00*	input-driven	*(B) 2.75*	*(B) 24.00*	*(B) 8.39*
		*medium*	→	*medium*	*medium*	*low-medium*
	*W1*	*(B) 1.94*	*output-driven*	(A) 2.50	(A) 19.00	(A) 10.00
		*low-medium*	←	low-medium	low-medium	low-medium
	*W1* *(new)*	*(B) 1.94*	input-driven	*(B) 2.42*	*(B) 19.30*	*(B) 10.93*
		*low-medium*	→	*low-medium*	*low-medium*	*low-medium*
Acute hospital care	*W2*	[0.33–10.55]				
Psychologist + nurses		(A) 5.50	input-driven	(B) 2.44	(B) 19.63	(B) 10.67
		medium	→	low-medium	low-medium	low-medium
Acute hospital care	*W3*	[1.84–24.40]				
Total professionals		(A) 14.00	input-driven	(B) 2.44	(B) 19.63	(B) 10.67
		medium	→	low-medium	low-medium	low-medium

**Fig. 6:**
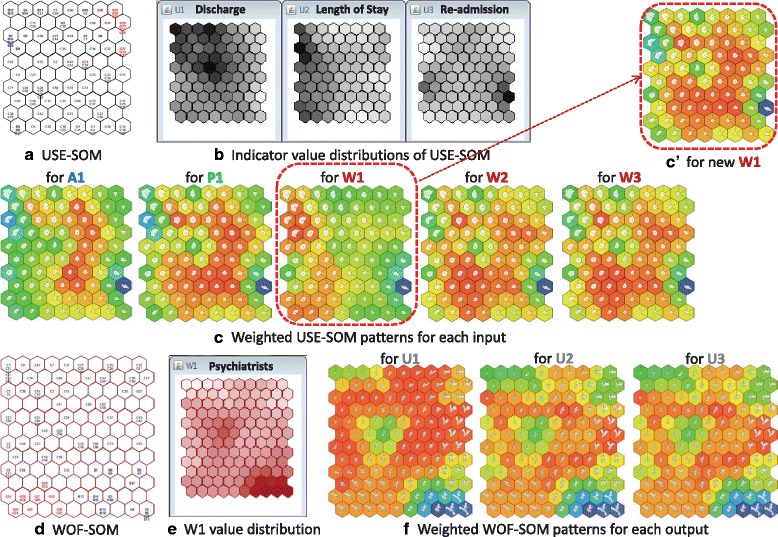
Visual analysis of the input and output indicator patterns by the SOMNet. **a** The output USE-SOM with areas. **b** The value planes of the output indicators, U1, U2 and U3. **c** The output pattern comparison using the input-driven analysis. **d** The input WOF-SOM with areas. **e** The value plane for the input indicator, W1. **f** The input pattern comparison using the output-driven analysis. **c'** The output USE-SOM pattern for the newly given W1 by the SOMNet analysis

In the input-driven analysis, the associational weights for the joint output of U1, U2 and U3 conditional on the inputs of A1, P1, W1, W2 and W3 were estimated in the output USE-SOM (Fig. 6a). The associational weight patterns of the USE-SOM for the given inputs were visually compared (Fig. 6c). The values of the output KPIs in the most weighted neurons (reddish region) are more related to the value given to an input KPI. Based on the region selection, the main output patterns driven by the given inputs were compared using the indicator value planes of the USE-SOM (Fig. 6b). The weighted USE-SOM patterns show the similar weight distributions except for the input value given to W1 by the prior expert knowledge. As also seen in Table 2, the ideal values (low-medium levels) of the three output KPIs (U1, U2 and U3) were estimated by the SOMNet for the given input KPIs of A1, P1, W2 and W3. However, the SOMNet estimated the medium level values of U1 and U2 and the low-medium level value of U3 for the given medium level value of W1. This showed that the number of psychiatrists (W1) at the given appropriate medium level value (‘3’) by the prior expert knowledge was not the most likely condition for the ideal utilisation of the acute hospital care services according to the SOMNet.

To identify the most likely condition of W1, an output-driven analysis was conducted to estimate the patterns of W1 in the WOF-SOM for the given ideal output values of U1, U2 and U3 (Table 2 and Fig. 6d). The reddish regions in the weighted WOF-SOM patterns commonly showed low-medium level values for W1 (Fig. 6e, f), indicating that the low-medium level value of W1 is the most likely condition for the ideal use of acute hospital care services. The low-medium value (‘1.94 ≈ 2’ psychiatrists in Table 2) estimated by the output-driven analysis was then applied to W1. Thus, the weighted USE-SOM pattern for the new W1 value shows the ideal utilisation pattern (Fig. 6c').

Using the SOMNet, the domain experts visually explored the KPI patterns and properties between different input and output datasets. Through the input- and output-driven analyses, the KPI relations were identified by comparing the values provided by the EbCA and the results obtained by the SOMNet. The analytical result of the SOMNet was interpreted as ‘the low-medium level of psychiatrists (W1) is the most likely related condition to the ideal use of acute care hospital services at the low-medium levels of discharge (U1), length of stay (U2) and re-admission (U3) across the mental health systems’. Based on the analytical interpretation in the mid-processing phase, the domain experts elicited new tacit knowledge. The evidence-based information obtained by the SOMNet with the EbCA confirmed the appropriateness of all the value ranges suggested by the domain experts except for the number of psychiatrists assigned to acute wards. This implies that reducing the value of W1 in the knowledge discovery process guides further analysis to examine the impact of W1 on service utilisation. The relevant decision-making could imply this information on the specific resource planning.

### Feasibility evaluation results

**Fig. 7:**
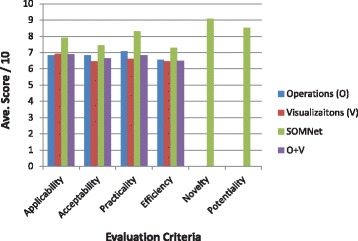
The feasibility results of the decision support systems model using the SOMNet approach. The average scores of the four feasibility evaluation dimensions are compared between the SOMNet and the other operation and visualisation approaches previously used in mental health studies. Two other dimensions (novelty and potentiality) are used separately to assess the SOMNet approach

The results of the model validation and usability have been measured by its feasibility and TRL [27] (described in Fig. 7 according to four feasibility domains). The ‘applicability’ was rated high (8/10), while the prior approaches received a slightly lower rating (7/10). Using the SOMNet, the domain experts obtained meaningful information that was relevant to their knowledge discovery and decision-making. The SOMNet has been accepted as an effective approach allowing the domain experts to be incorporated in exploring and processing evidence-based information without complicated processes. Using the interactive visual data mining of the SOMNet, its ‘acceptability’ was rated higher than the other visualisation tools in attaining the specified study objectives. The ‘practicality’ of the SOMNet approach was also rated higher, compared with the other visualisation tools, for analysing mental health problems. The unique visualisation techniques in the SOMNet allowed better incorporation of the domain experts in the mid-processing phase from modelling data to eliciting tacit knowledge through interactive visual data mining. The ‘feasibility efficiency’ of the SOMNet was rated higher than the other approaches since it produced correct and useful information in terms of both qualitative and quantitative measures.

The ‘novelty’ and ‘potentiality’ of the SOMNet approach for DSS were separately assessed to determine how the domain experts have experienced and prospected the SOMNet application for DSS. The ‘novelty’ was rated very high (approximately 9/10) (Fig. 7), since it was a new data analysis experience for the domain experts. The ‘potentiality’ was also rated very high (approximately 8.5/10), motivating the domain experts to use the SOMNet approach for DSS when conducting further data analysis in various mental health studies. The feasibility results indicated that the SOMNet approach reached level 7 of the TRL (system prototype demonstration in operational environment).

## Discussion

Health systems engineering has been adopted in the United States to analyse healthcare systems, to provide recommendations for reducing healthcare waste and to improve its overall efficiency. It connects systems engineering principles and best practices with clinical expertise for DSS to improve understanding of the interactions among agents (clinicians, patients, families and other stakeholders), processes (institutional, regulatory, professional ethics, etc.) and technologies (medical devices and instrumentation), and to formulate innovations and better outcomes [42].

To our knowledge, this is the first study that provides an integrated approach to DSS in mental health, following a systems thinking approach [1] and resulting in actual use by governmental agencies. This study is based on a long-term collaboration between a research network led by the PSICOST Research Association and several regional departments of health in Spain. This collaboration has facilitated (1) the design of a systems-based framework, (2) the exploration of different methodological approaches to the use of DSS for planning, and (3) the incorporation of domain experts at all levels of data processing. This innovative study should be understood in the context of two emerging disciplines, namely healthcare delivery science [6] and healthcare ecosystems research [43].

The use of the SOMNet combined with the EbCA has overcome some of the problems of previous modelling techniques for analysing complex mental health systems, especially in the mid-processing of the data. The prior exclusion of domain experts in the mid-processing phase created a gap between experts and analysts in complex data analysis and made it difficult for domain experts to reach an evidence-informed decision [44]. The interactive visual data mining function of the SOMNet has incorporated domain experts (by the EbCA), and the SOMNet application has shown some relevant advantages resulting from the incorporation of domain experts into the data analysis process. The advantages are (1) that this model is free from the selection of significant variables and model fitting; (2) it identifies all multi-level patterns of complex data; and (3) that its analytical results are evidential and insightful, and motivate domain experts to improve their knowledge for decision support.

The SOMNet approach allowed domain experts to guide data analysis based on their interests and to understand the results better. The domain experts were enabled to identify and process the catchment area outliers according to their connectivity with the rest of the system. For example, highly rural areas in Catalonia and areas with a predominantly private model of management in Gipuzkoa could be excluded from the system analysis, while other outlier urban areas in Catalonia required adjustment of their KPIs to be incorporated into the model. The SOMNet also facilitated the identification and explanation of the global and local system patterns at macro-level and meso-level analysis, respectively, across different systems. Finally, the SOMNet examined the input and output relationships of the KPIs, confirming the appropriateness of the assumptions provided by prior expert knowledge and questioning one of the KPIs assessed (workforce capacity of psychiatrists in acute hospital care). These tasks are critical for knowledge-informed planning in mental health.

Additionally, this study provides a detailed analysis of the external validity (feasibility) of the SOMNet and its TRL [27]. It is important to incorporate an end-user appraisal, which allowed a technology level 7 to be reached (system prototype demonstration in operational environment). The domain experts found that the SOMNet approach allowed them to think differently and facilitated an agile view of the data patterns and behaviours. The interactive visual data mining of the SOMNet improved evidence-informed policy planning and showed potential for DSS. However, the EbCA [11] can be improved by adding a model assessment stage in its post-processing of data for further expert-guided data analysis.

A number of limitations should be noted. First, this combined approach introduces new concepts and techniques in health planning. Even though many of them have been extensively tested in other fields of knowledge, the use of systems thinking and combined modelling are in their infancy in mental health policy. Nevertheless, many of the functional aspects of this study have been previously published (e.g. [3, 12, 17, 45]). New contributions include the extension of the mid-processing phase of KDD or the feasibility analysis of the SOMNet approach within the context of the TRL of DSS in mental health care research. Longitudinal and corroboration studies are required to differentiate innovative discovery from the limitations on the validity and usability of this model. Regardless, the overall aim of this study is not precision, accuracy and/or causality but to increase the organisational learning of health planning agencies from an implementation sciences and integrated care perspective [46]. Second, the SOMNet visual tools were unfamiliar and challenging for the domain experts to interpret. Visualisation tools could be divided into (1) tools that permit users to construct customised visualisation but require advanced technical skills and (2) tools that allow only predefined visualisation without advanced technical skills such as graphs [47, 48]. The SOMNet visualisation tools allowed the domain experts to explore, manipulate and interpret the analytical hypotheses and results. However, it required a significant level of technical skills for their interaction, since the way of visualising information on the hexagonal grid map was very new and unfamiliar to the domain experts. Third, the number of domain experts that participated in the feasibility analysis was small. A challenge for assessing the actual usability of this DSS is the small number of end-users with sufficient knowledge of the specific regional systems to provide a critical assessment of the new tools. This substantially limits the robustness of the external validation of the DSS and requires further research in other countries and by other groups. Finally, other resource inputs, such as sociodemographic indicators and the use of day care, costs and patient-reported outcomes, should be incorporated to improve the accuracy and usability of the DSS. Similarly, the use of this model for the analysis of longitudinal data on the health systems and its application to the analysis of health interventions are relevant topics for future research.

## Conclusions

Obtaining scientific evidence by incorporating domain experts through empirical study in a real-world context is important in mental health research for knowledge discovery and decision support. Since mental health systems are highly complex, the underlying system patterns and behaviours have been hardly understood by existing data analysis approaches in the mid-processing of data (data formation, data mining and results evaluation). In this paper, we developed a DSS model for interactive visual data mining by combining the SOMNet approach with the EbCA approach for the mental health systems analysis in the mid-processing phase of KDD. The SOMNet was applied to the analysis of healthcare patterns and indicators of three different regional mental health systems in Spain. The relevant analytical goals defined in the study were achieved by incorporating the domain experts in the SOMNet analysis. The domain experts were able to (1) identify and process the catchment area outliers; (2) identify and explain the global and local system patterns across the different systems; and (3) identify and examine the input and output relationships of the key performance indicators, which guided further analysis for evidence-informed knowledge discovery. The domain experts also evaluated the feasibility of the SOMNet approach for DSS based on its analytical processes and outcomes. The feasibility evaluation results showed that the SOMNet approach is relevant and useful for analysing highly complex mental health systems and improving evidence-informed knowledge discovery. The technology used in this study (the system prototype) demonstrated potential for DSS implementation in the operational environment (TRL 7).

This health systems engineering technique (the SOMNet) combined with expert knowledge (the EbCA) is an innovative and powerful method to address complex questions in health systems research and planning in a real-world context.

## Additional files

Additional file 1: Information on the model validation process. This document provides information about ethics approval for the expert-based model validation process, the definition of feasibility dimensions, the feasibility checklist and the technology readiness levels (TRL). (PDF 648 kb)

Additional file 2: Labelled small mental health areas in Spain. The small mental health areas in Biscay, Gipuzkoa and Catalonia systems in Spain are labelled using the system initial and Arabic numbers to be simplified in the data analysis. (PDF 211 kb)

Additional file 3: Geographical maps of the mental health systems in Spain. The labelled small mental health areas in the three systems are shown on the geographical maps of the Basque Country and Catalonia in Spain. (PDF 519 kb)

Additional file 4: Information on the DESDE-LTC classification system. This document provides information on the use of DESDE-LTC system for mental health service coding and mapping to develop the Atlas of Mental Health Care. (PDF 551 kb)

## Abbreviations

ANN: Artificial neural network
AVA: Availability
DEA: Data envelopment analysis
DSS: Decision support systems
EbCA: Expert-based collaborative analysis
KDD: Knowledge discovery in database
KPI: Key performance indicator
MMS: Minimum metadata set
PLA: Placement
SOM: Self-organising map
SOMNet: Self-organising map network
TRL: Technology readiness level
USE: Utilisation
WOF: Workforce
