# Supplementary material for: Use of the self-organising map network (SOMNet) as a decision support system for regional mental health planning
Source: Health Res Policy Syst. 2018 Apr 25;16:35. doi: 10.1186/s12961-018-0308-y (PMC5922302; doi:10.1186/s12961-018-0308-y)
Supplement: Supplementary file 1 — Information on the model validation process. This document provides information about ethics approval for the expert-based model validation process, the definition of feasibility dimensions, the feasibility checklist and the technology readiness levels (TRL). (PDF 648 kb) [file 12961_2018_308_MOESM1_ESM.pdf]

Information on the model validation process

The Ethics Approval

This study was approved by the Human Research Ethics Committee (HRECs) at The University of Sydney, Australia. The ethics approval was on the project titled, “A study on the feasibility of SOMNet for improving evidence-informed health policy planning” (Approval Number: 2017/051) for the domain expert participation to provide their feedback on the model evaluation.

The definitions of six feasibility dimensions

- Applicability (Relevance): The degree to which the model processes and outcomes have a likelihood of being meaningful for data analysis and relevant to decision making.
- Acceptability (Effectiveness): The degree to which the model provides the desired (e.g. user-friendly) processes and outcomes to the experts in attaining the specified objectives.
- Practicality (Usefulness): The degree to which the model allows the experts to complete a set of tasks and to achieve specific goals for data analysis and knowledge discovery.
- Efficiency: The degree to which the model produces correct and useful information in terms of quantity and quality without complicated processes (process cost-effective).
- Novelty: The degree to which the model is new, different and interesting for data mining.
- Potentiality: The degree to which the model motivates its use for further study developments.

The value range format for rating the feasibility checklist

Qualitative	Quantitative
low	0 – 1.99
Low-medium	2 – 3.99
medium	4 – 5.99
Medium-high	6 – 7.99
high	8 – 10

The feasibility checklist

How do you value the following evaluation criteria on the previous approaches and the SOMNet approach for discovering knowledge and decision making in health planning and policy?

For each item below, please indicate the extent to how you value the model based on the question statement using the scale provided. Please Mark with ‘X’ and give a score (0-10) for your rating.

The previous approaches (e.g. Monte Carlo DEA)

**1.**	The practical ***usefulness*** of using other *operations research models*	**LOW**	**LOW MEDIUM**	**MEDIUM**	**MEDIUM HIGH**	**HIGH**
	Score: 0 (very low) - 10 (very high)					

**2.**	The practical ***efficiency*** of using other *operations research models*	**LOW**	**LOW MEDIUM**	**MEDIUM**	**MEDIUM HIGH**	**HIGH**
	Score: 0 (very low) - 10 (very high)					

**3.**	The practical ***effectiveness*** of using other *operations research models*	**LOW**	**LOW MEDIUM**	**MEDIUM**	**MEDIUM HIGH**	**HIGH**
	Score: 0 (very low) - 10 (very high)					

**4.**	The practical ***relevance*** of using other *operations research models*	**LOW**	**LOW MEDIUM**	**MEDIUM**	**MEDIUM HIGH**	**HIGH**
	Score: 0 (very low) - 10 (very high)					

**5.**	The practical ***usefulness*** of using other *visualization tools*	**LOW**	**LOW MEDIUM**	**MEDIUM**	**MEDIUM HIGH**	**HIGH**
	Score: 0 (very low) - 10 (very high)					

**6.**	The practical ***efficiency*** of using other *visualization tools*	**LOW**	**LOW MEDIUM**	**MEDIUM**	**MEDIUM HIGH**	**HIGH**
	Score: 0 (very low) - 10 (very high)					

**7.**	The practical ***effectiveness*** of using other *visualization tools*	**LOW**	**LOW MEDIUM**	**MEDIUM**	**MEDIUM HIGH**	**HIGH**
	Score: 0 (very low) - 10 (very high)					

**8.**	The practical ***relevance*** of using other *visualization tools*	**LOW**	**LOW MEDIUM**	**MEDIUM**	**MEDIUM HIGH**	**HIGH**
	Score: 0 (very low) - 10 (very high)					

The SOMNet approach

**1.**	The practical ***usefulness*** of using the *SOMNet approach*	**LOW**	**LOW MEDIUM**	**MEDIUM**	**MEDIUM HIGH**	**HIGH**
	Score: 0 (very low) - 10 (very high)					

**2.**	The practical ***efficiency*** of using the *SOMNet approach*	**LOW**	**LOW MEDIUM**	**MEDIUM**	**MEDIUM HIGH**	**HIGH**
	Score: 0 (very low) - 10 (very high)					

**3.**	The practical ***effectiveness*** of using the *SOMNet approach*	**LOW**	**LOW MEDIUM**	**MEDIUM**	**MEDIUM HIGH**	**HIGH**
	Score: 0 (very low) - 10 (very high)					

**4.**	The practical ***relevance*** of using the *SOMNet approach*	**LOW**	**LOW MEDIUM**	**MEDIUM**	**MEDIUM HIGH**	**HIGH**
	Score: 0 (very low) - 10 (very high)					

**5.**	The practical ***novelty*** of using the *SOMNet approach*	**LOW**	**LOW MEDIUM**	**MEDIUM**	**MEDIUM HIGH**	**HIGH**
	Score: 0 (very low) - 10 (very high)					

**6.**	The practical ***potentiality*** of using the *SOMNet approach*	**LOW**	**LOW MEDIUM**	**MEDIUM**	**MEDIUM HIGH**	**HIGH**
	Score: 0 (very low) - 10 (very high)					

**7.**	Do you have any ***comments*** or ***suggestions*** for improving the *SOMNet approach* in conducting your data analysis?	

Technology Readiness Level (TRL)

G. Technology readiness levels (TRL). HORIZON 2020 – Work Programme 2014-2015 General Annexes, Extract from Part 19 - Commission Decision C4995 (2014)

Technology Readiness Level	Description
TRL 1.	basic principles observed
TRL 2.	technology concept formulated
TRL 3.	experimental proof of concept
TRL 4.	technology validated in lab
TRL 5.	technology validated in relevant environment (industrially relevant environment in the case of key enabling technologies)
TRL 6.	technology demonstrated in relevant environment (industrially relevant environment in the case of key enabling technologies)
TRL 7.	system prototype demonstration in operational environment
TRL 8.	system complete and qualified
TRL 9.	actual system proven in operational environment (competitive manufacturing in the case of key enabling technologies; or in space)
