# Supplementary material for: Use of the self-organising map network (SOMNet) as a decision support system for regional mental health planning
Source: Health Res Policy Syst. 2018 Apr 25;16:35. doi: 10.1186/s12961-018-0308-y (PMC5922302; doi:10.1186/s12961-018-0308-y)
Supplement: Supplementary file 2 — Labelled small mental health areas in Spain. The small mental health areas in Biscay, Gipuzkoa and Catalonia systems in Spain are labelled using the system initial and Arabic numbers to be simplified in the data analysis. (PDF 211 kb) [file 12961_2018_308_MOESM2_ESM.pdf]

Data	Area Name (Biscay)	Data	Area Name (Gipuzkoa)
B1	Ajuriaguerra	G20	Alto Deba-Arrasate
B2	Barakaldo	G21	Amara
B3	Basauri	G22	Andoain
B4	Bermeo	G23	Azpeitia
B5	Derio	G24	Beasain
B6	Durango	G25	Eguia
B7	Erandio	G26	Eibar
B8	Ercilla	G27	Irun
B9	Etxaniz	G28	Ondarreta
B10	Galdakao	G29	Renteria
B11	Gernika	G30	Tolosa
B12	Ortuella	G31	Zarautz
B13	Otxarkoaga	G32	Zumarraga
B14	Portugalete		
B15	Santurtzi		
B16	Sestao		
B17	Uribe		
B18	Zalla		
B19	**Rekalde**		

(a) The Basque Country

Data	Area Name	Data	Area Name	Data	Area Name	Data	Area Name
C1	CSMA Alt Empordà	C21	CSMA Esplugues de Llobregat	C41	CSMA Mollet	C61	CSMA Segarra de Cervera
C2	CSMA Alt Penedès	C22	CSMA Garraf	C42	CSMA Mora d'Ebre	C62	CSMA Selva Marítima
C3	CSMA Amposta	C23	CSMA Gavà	C43	CSMA Nou Barris Nord	C63	**CSMA Seu d'Urgell**
C4	CSMA Anoia	C24	CSMA Gironès - Pla De L'Estany	C44	CSMA Nou Barris Sud	C64	**CSMA Sort**
C5	CSMA Badalona 1	C25	**CSMA Gràcia**	C45	CSMA Olot-Garrotxa	C65	CSMA Tarragona Nord
C6	CSMA Badalona 2	C26	CSMA Guinardó	C46	CSMA Osona	C66	CSMA Tarragona Sud
C7	CSMA Badia	C27	CSMA Horta	C47	CSMA Poble Sec/Montjuïc	C67	CSMA Tàrrega
C8	CSMA Bages-Manresa	C28	CSMA Hospitalet 1	C48	CSMA Reus	C68	CSMA Terrassa 1
C9	CSMA Baix Empordà	C29	CSMA Hospitalet 2 (ICS)	C49	CSMA Ripollès	C69	CSMA Terrassa 2
C10	CSMA Balaguer	C30	CSMA La Mina	C50	CSMA Rubí	C70	CSMA Tortosa
C11	CSMA Berga	C31	CSMA La Selva Interior	C51	CSMA Sabadell 1	C71	CSMA Tremp
C12	CSMA Borges Blanques	C32	CSMA Les Corts	C52	CSMA Sabadell 2	C72	CSMA Vallès Oriental
C13	CSMA Castelldefels	C33	CSMA Lleida	C53	CSMA Sant Andreu	C73	CSMA Valls
C14	CSMA Cerdanyola-Ripollet-Montcada	C34	CSMA Maragall	C54	CSMA Sant Boi	C74	**CSMA Vielha**
C15	CSMA Ciutat Vella	C35	**CSMA Maresme Centre/ Mataró**	C55	CSMA Sant Cugat		
C16	CSMA Cornellà	C36	CSMA Maresme Nord	C56	CSMA Sant Feliu de Llobregat		
C17	**CSMA Eixample Dreta**	C37	CSMA Maresme Sud	C57	CSMA Sant Martí Nord		
C18	CSMA Eixample Esquerra	C38	CSMA Martí i Julià	C58	CSMA Sant Martí Sud		
C19	CSMA El Prat de Llobregat	C39	CSMA Martorell	C59	CSMA Sants		
C20	CSMA El Vendrell	C40	CSMA Mollerussa	C67	CSMA Sarrià-Sant Gervasi		

(b) Catalonia
