# Supplementary figures and images for: Use of the self-organising map network (SOMNet) as a decision support system for regional mental health planning
Source: Health Res Policy Syst. 2018 Apr 25;16:35. doi: 10.1186/s12961-018-0308-y (PMC5922302; doi:10.1186/s12961-018-0308-y)

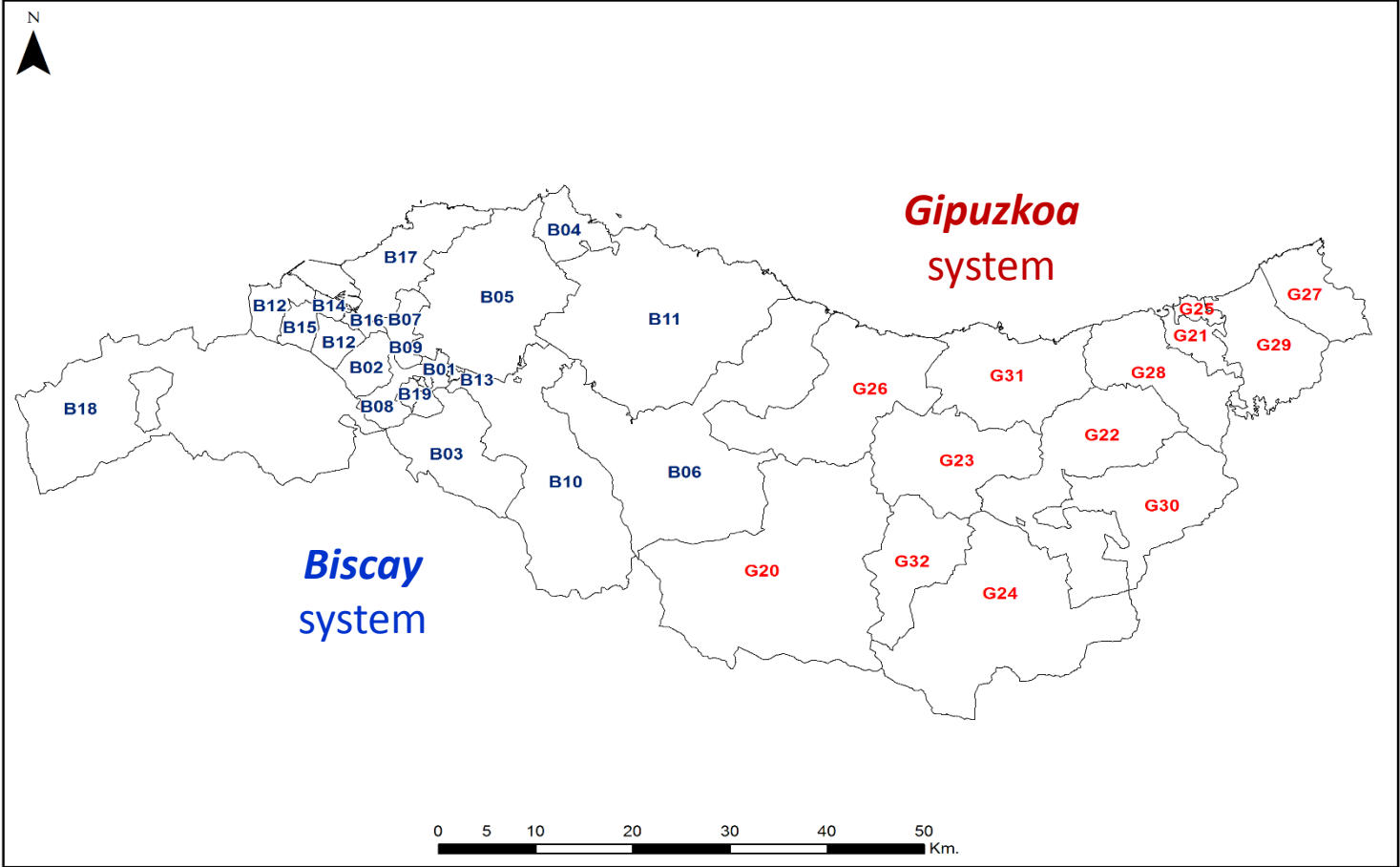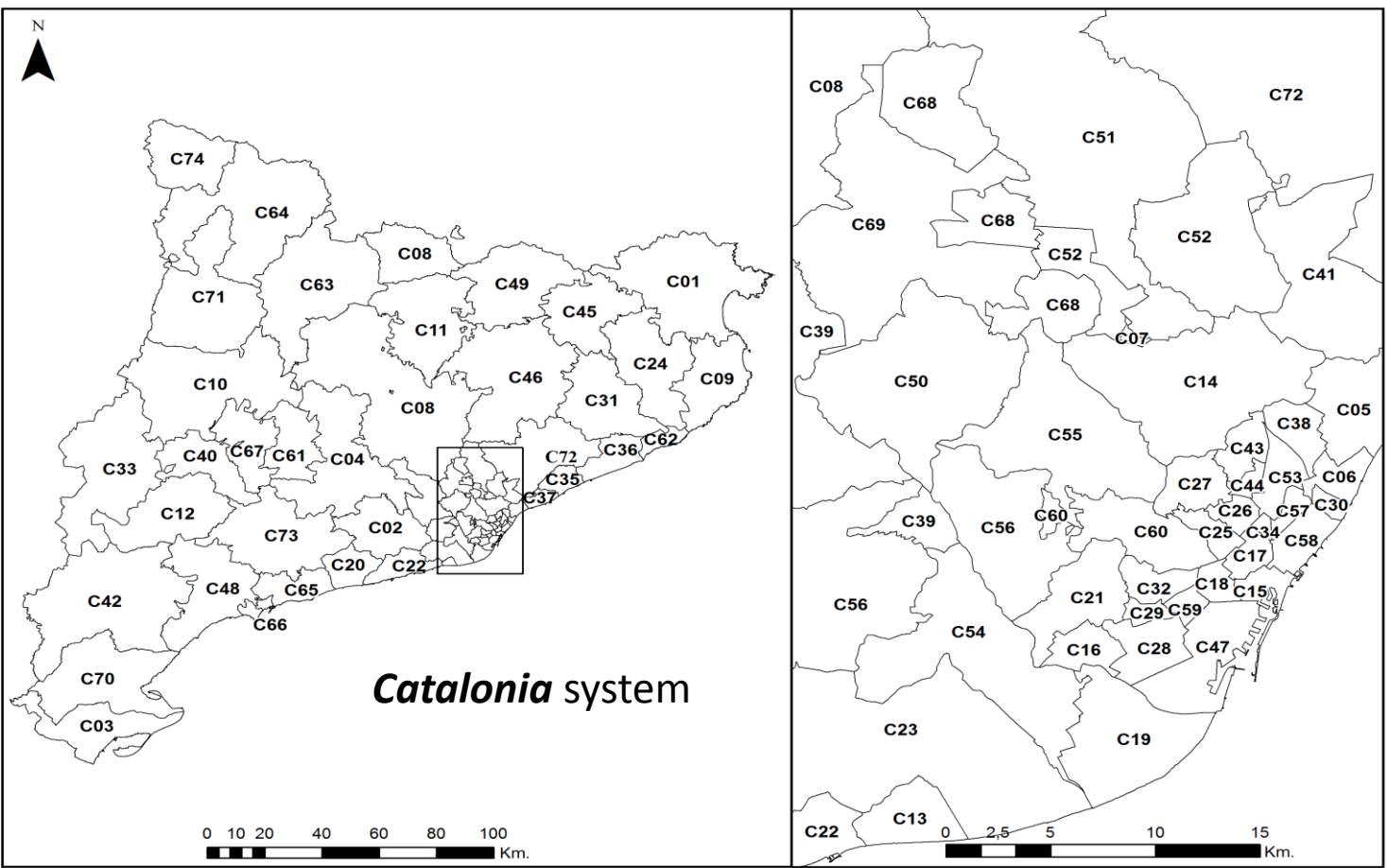

Supplement: Supplementary file 3 — Geographical maps of the mental health systems in Spain. The labelled small mental health areas in the three systems are shown on the geographical maps of the Basque Country and Catalonia in Spain. (PDF 519 kb) [file 12961_2018_308_MOESM3_ESM.pdf]
