# Supplementary material for: Use of the self-organising map network (SOMNet) as a decision support system for regional mental health planning
Source: Health Res Policy Syst. 2018 Apr 25;16:35. doi: 10.1186/s12961-018-0308-y (PMC5922302; doi:10.1186/s12961-018-0308-y)
Supplement: Supplementary file 4 — Information on the DESDE-LTC classification system. This document provides information on the use of DESDE-LTC system for mental health service coding and mapping to develop the Atlas of Mental Health Care. (PDF 551 kb) [file 12961_2018_308_MOESM4_ESM.pdf]

USE OF DESDE-LTC FOR MAPPING, COMPARATIVE ANALYSIS OF SERVIVE PROVISION, AND MODELLING

A) Multi-step mental health service coding and mapping process using DESDE-LTC.

Step 1 - Local identification of all the services available in the system and identification of the care teams or BSICs in every service; Step 2 - coding all the BSICs identified using an international taxonomy based on activity and not on the names of the services (main types of care - MTC); Step 3 - Additional coding of the target population and qualifiers; Step 4 - geolocation of services in relation to local morbidity and aggregation of data to the regional level to prepare the Atlas of Mental Health Care

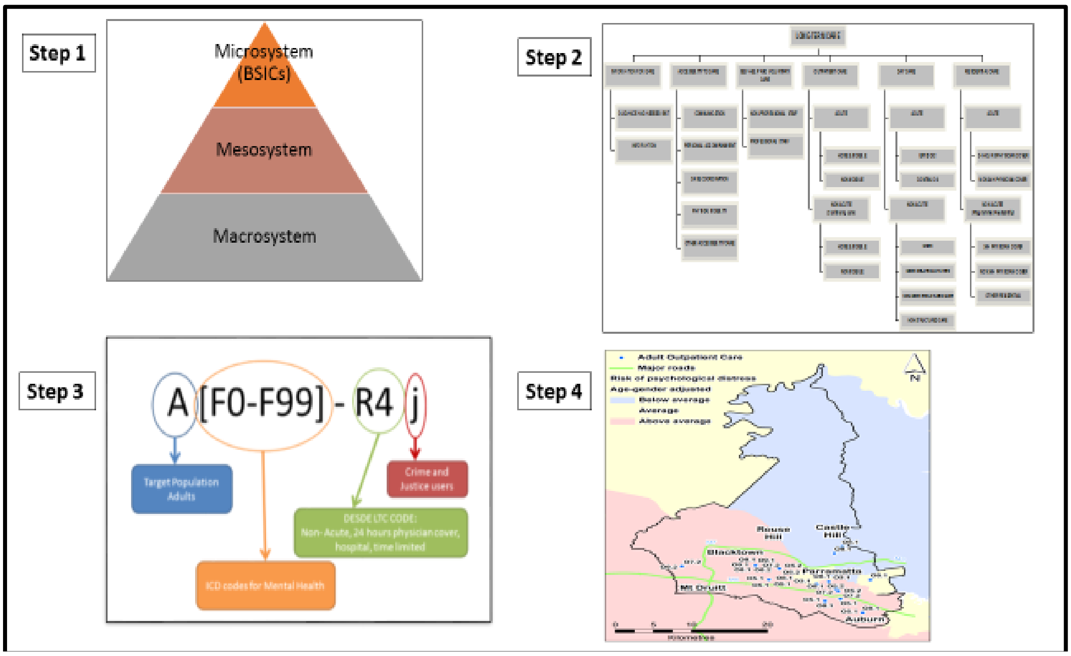

B) Development of the Atlas of Mental Health Care

Step 5 - Geo-spatial representation of the socio-demographics, population, prevalence, and utilisation data; Step 6 - Analysis of the Balance of Care (e.g. hospital vs. outpatient care, health vs. social care); Step 7 - Availability of services by their statutory name and by MTC (DESDE-LTC), places and beds (placement capacity) and professionals (workforce capacity); Step 8 - patterns of care provision and comparison with other jurisdictions (regional, state, national and international)

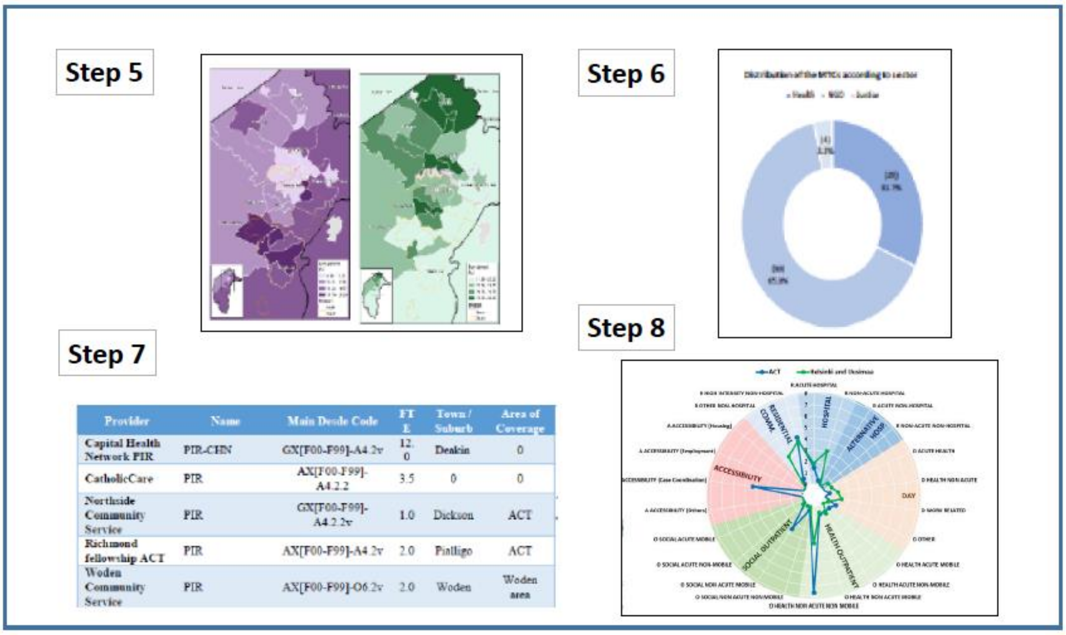

Provider	Name	Main Desde Code	FTE	Town / Suburb	Area of Coverage
Capital Health Network PIR	PIR-CHN	GX[F00-F99]-A4.2v	12.0	Deakin	0
CatholicCare	PIR	AX[F00-F99]-A4.2.2	3.5	0	0
Northside Community Service	PIR	GX[F00-F99]-A4.2.2v	1.0	Dickson	ACT
Richmond fellowship ACT	PIR	AX[F00-F99]-A4.2v	2.0	Pialligo	ACT
Woden Community Service	PIR	AX[F00-F99]-O6.2v	2.0	Woden	Woden area
